# Genetic Variants Associated with Chronic Kidney Disease in a Spanish Population

**DOI:** 10.1038/s41598-019-56695-2

**Published:** 2020-01-10

**Authors:** Zuray Corredor, Miguel Inácio da Silva Filho, Lara Rodríguez-Ribera, Antonia Velázquez, Alba Hernández, Calogerina Catalano, Kari Hemminki, Elisabeth Coll, Irene Silva, Juan Manuel Diaz, José Ballarin, Martí Vallés Prats, Jordi Calabia Martínez, Asta Försti, Ricard Marcos, Susana Pastor

**Affiliations:** 1 Grup de Mutagènesi, Departament de Genètica i de Microbiologia, Edifici C, Universitat Autònoma de Barcelona, Bellaterra, Cerdanyola del Vallès Spain; 2 Division of Molecular Genetic Epidemiology, German Cancer Research Center (DKFZ), Heidelberg, Germany; 3 Consortium for Biomedical Research in Epidemiology and Public Health (CIBERESP), Carlos III Institute of Health, Madrid, Spain; 4 Fundació Puigvert, Nephology Department, Barcelona, Spain; 5 Hospital Josep Trueta, Girona, Spain

**Keywords:** Genetic association study, End-stage renal disease

## Abstract

Chronic kidney disease (CKD) patients have many affected physiological pathways. Variations in the genes regulating these pathways might affect the incidence and predisposition to this disease. A total of 722 Spanish adults, including 548 patients and 174 controls, were genotyped to better understand the effects of genetic risk loci on the susceptibility to CKD. We analyzed 38 single nucleotide polymorphisms (SNPs) in candidate genes associated with the inflammatory response (interleukins *IL-1A*, *IL-4*, *IL-6*, *IL-10*, *TNF-α*, *ICAM-1)*, fibrogenesis (*TGFB1*), homocysteine synthesis (*MTHFR*), DNA repair (*OGG1*, *MUTYH*, *XRCC1*, *ERCC2*, *ERCC4*), renin-angiotensin-aldosterone system (*CYP11B2*, *AGT*), phase-II metabolism *(GSTP1*, *GSTO1*, *GSTO2)*, antioxidant capacity (*SOD1*, *SOD2*, *CAT*, *GPX1*, *GPX3*, *GPX4*), and some other genes previously reported to be associated with CKD (*GLO1*, *SLC7A9*, *SHROOM3*, *UMOD*, *VEGFA*, *MGP*, *KL*). The results showed associations of *GPX1*, *GSTO1*, *GSTO2*, *UMOD*, and *MGP* with CKD. Additionally, associations with CKD related pathologies, such as hypertension (*GPX4*, *CYP11B2*, *ERCC4*), cardiovascular disease, diabetes and cancer predisposition (*ERCC2*) were also observed. Different genes showed association with biochemical parameters characteristic for CKD, such as creatinine (*GPX1*, *GSTO1*, *GSTO2*, *KL*, *MGP*), glomerular filtration rate (*GPX1*, *GSTO1*, *KL*, *ICAM-1*, *MGP*), hemoglobin (*ERCC2*, *SHROOM3*), resistance index erythropoietin (*SOD2*, *VEGFA*, *MTHFR*, *KL*), albumin (*SOD1*, *GSTO2*, *ERCC2*, *SOD2*), phosphorus (*IL-4*, *ERCC4 SOD1*, *GPX4*, *GPX1*), parathyroid hormone (*IL-1A*, *IL-6*, *SHROOM3*, *UMOD*, *ICAM-1*), C-reactive protein (*SOD2*, *TGFB1*,*GSTP1*, *XRCC1)*, and ferritin (*SOD2*, *GSTP1*, *SLC7A9*, *GPX4*). To our knowledge, this is the second comprehensive study carried out in Spanish patients linking genetic polymorphisms and CKD.

## Introduction

Chronic kidney disease (CKD) is becoming a major public health problem worldwide. CKD is defined as a progressive loss of renal function, measured by a decline in glomerular filtration rate (GFR < 60 mL/min/1.73 m^2^)^1^, which is typically associated with irreversible pathological changes within the kidney. This pathology has a complicated interrelationship with other diseases^2,3^. Diabetes (DM) and hypertension (HT) are the primary risk factors for CKD^4^, and CKD is also associated with cardiovascular morbidity and mortality^5,6^, even in early stages and in young patients^7^.

CKD patients are also characterized by a high genomic instability^8–11^. This instability could be translated to high levels of genetic damage measured by the incidence of chromosomal damage (micronuclei) when their cells are challenged with ionizing radiation^12^ and could be either the cause or the consequence of renal pathologies. In addition, it has been observed that CKD patients repair less efficiently DNA damage^13^.

CKD patients present increased levels of C-reactive protein (CRP), which is indicative of an inflammatory status^12,14,15^. Oxidative stress is also a characteristic usually shown by CKD patients^16–19^. Variants in genes regulating such different pathways may affect CKD incidence and/or its progression. In this context recent genome-wide association studies (GWASs) on large European populations have identified novel genetic risk single-nucleotide polymorphisms (SNPs) associated with different CKD related pathologies like hypertension^20^, coronary artery disease^21^, subclinical vascular disease^22^, and kidney functional traits in CKD patients^23–25^. Other studies have shown an overlap between genetic variants underpinning kidney traits and cardiovascular pathologies^25^.

Aiming to determine possible associations between allelic variants and susceptibility to CKD, we selected and genotyped 38 SNPs from 31 candidate genes related directly to CKD and to the additional diseases (mainly hypertension, diabetes and inflammation, among others) in a Spanish population.

## Results

### Population

Table 1 shows some general characteristics of the individuals under study. Among CKD patients there were more men than women, reflecting the well-known higher incidence of CKD in males. As expected, statistically significant differences were observed between cases and controls for different parameters related to the pathology. The differences were highly significant (*P* < 0.001) for the levels of age, creatinine, glomerular filtration rate, hemoglobin, albumin, parathyroid hormone, C-reactive protein, uric acid, proteinuria, and urea. The number of samples included in some comparisons is too small to allow definite conclusions. The CKD patients show the main characteristics of the CKD populations as are reflexed in Table 1. The group of patients have more individuals affected by hypertension (91.5% *vs* a 29% in the control group), and with *diabetes mellitus* (DM) (32% *vs* a 7.5% in the control group). Regarding the cardiovascular disease (CVD), it could be said that 45% of the CKD patients presented CVD. Unfortunately, for the control group, we only have 53% of the answers, and of these nobody present CVD,

**Table 1:** Description of the study population. Differences in biochemical and clinical parameters of the studied groups are indicated.

Characteristic^a^	Controls N = 174 (N) mean ± SD	Cases N = 548 (N) mean ± SD
Age (years)	(169) 56.06 ± 15.31	(547) 66.34 ± 13.40***
Gender (men/women)	105 (61%)/67 (39%) %0/67	338 (62%)/210 (38%)
Creatinine (45–80 µmol/L)^a^	(169) 69.86 ± 14.36	(184) 200.95 ± 92.92***
Glomerular Filtration Rate (>60 mL/min/1.75 m^2^)	(169) 86.82 ± 6.98	(184) 31.22 ± 13.69***
Erythropoietin/month (µg Darbepoetin/month)	ND	(400) 207.61 ± 689.91
Erythropoietin Resistance Index (<10)	ND	(379) 13.50 ± 37.45
Hemoglobin (120–160 g/L)	(96) 144.18 ± 11.96	(539) 128.41 ± 18.23***
Glucose (4–5.8 µmol/L)	(113) 5.86 ± 1.93	(412) 5.60 ± 1.85*
Cholesterol (3.20–5.20 mmol/L)	(86) 5.22 ± 1.04	(542) 4.48 ± 1.16**
Triglycerides (0.30–1.40 mmol/L)	(84) 1.34 ± 1.19	(542) 1.50 ± 0.79**
Albumin (37–47 g/L)	(66) 44.06 ± 4.28	(405) 40.70 ± 4.52***
Calcium (2.1–2.55 mmol/L)	(70) 2.33 ± 0.11	(542) 2.29 ± 0.25
Phosphorus (0.8–1.3 mmol/L)	(68) 1.07 ± 0.14	(542) 1.30 ± 0.40**
Parathyroid hormone (7–53 ng/L)	(25) 61.23 ± 23.58	(401) 190.19 ± 179.09***
Ferritin (25–250 µg/L)	(3) 88.56 ± 49.32	(325) 245.87 ± 257.66
C-Reactive Protein (<10 mg/L)	(13) 2.80 ± 3.21	(362) 10.16 ± 19.40*
Uric acid (210–420 µmol/L)	(98) 302.54 ± 85.50	(286) 385.80 ± 109.60***
Proteinuria/24 h (<0.15 g/L)	(116) 0.14 ± 0.73	(165) 0.81 ± 1.43***
Urea (2.5–7 Mmol/L)	(56) 5.67 ± 1.63	(179) 15.32 ± 6.88***
Hba1c glycosylated hemoglobin (<5.7%)	(10) 5.18 ± 2.43	(158) 4.80 ± 1.62
Fibrinogen (2–4 µmol/L)	(2) 4.35 ± 1.11	(91) 4.47 ± 0.98
Hypertension (yes/no)	27 (29%)/66 (71%)	498 (91.5%)/46 (8.5%)***
*Diabetes Mellitus* (yes/no)	7 (7.5%)/86 (92.5%)	174 (32%)/370 (68%)***

Mann-Whitney test; cases vs controls; ***P < 0.001, **P < 0.01, *P < 0.05. ND: no data. ^a^normal values are shown in parentheses.

The slight differences in the numbers of patients reported for different parameters are due to their absence in the questionnaires, or to failure in the corresponding analysis.

### SNPs associated with CKD

General information about the 38 SNPs included in the study, with their allelic frequencies and their location in the genome is described in Table 2. As indicated, we used alternative SNPs in strong linkage disequilibrium (LD) with the selected one, when no assay corresponding to the originally selected SNP was available.

**Table 2:** Description of the SNPs selected for this study.

Function group	Gene	SNP original	SNP alternative	LD (r^2^)	Chr	Position (NCBI dbSNP GRCh38)	Consequence of the original SNP*	Minor allele	Major allele	Minor allele frequency (NCBI dbSNP)
	*GLO1*	rs386572987	rs4746	1.00	6	38682852	missense^a^ Glu111Ala	G	T	0.2873
Associated with CKD	*SLC7A9*	rs12460876			19	32865985	intron variant	C	T	0.4235
	*SHROOM3*	rs17319721			4	76447694	intron variant	A	G	0.2238
	*UMOD*	rs12917707			16	20356368	66 bp 5′ of UMOD	T	G	0.0982
	*VEGFA*	rs881858			6	43838872	13 kb 3′ of RP11–344J7.2	G	A	0.3626
	*IL-1A*	rs1800587	rs17561	0.99	2	112779646	5′-UTR	A	C	0.2175
Cytokines^c^	*IL-4*	rs2243250	rs2070874	0.99	5	132674018	2KB 5′IL-4 ^a^	T	C	0.4012
	*IL-6*	rs1800795	rs1800797	0.97	7	22726602	intron variant	A	G	0.1382
	*IL-10*	rs1800896			1	206773552	1.1 kb 5′ of IL10	C	T	0.2722
	*TNF-α*	rs1800629			6	31575254	312 bp 5′ of TNF	A	G	0.0903
	*ICAM-1*	rs5498			19	10285007	missense Glu469lys	G	A	0.3588
Renin-angiotensin-aldosterone^c^	*CYP11B2*	rs1799998			8	142918184	340 bp 5′ of CYP11B2	G	A	0.3472
	*AGT*	rs5050			1	230714140	5′ UTR	G	T	0.1759
Fibrogenesis^c^	*TGFB1*	rs1800470			19	41353016	Missense Pro10Leu	G	A	0.4547
		rs1800468			19	41354682	3′UTR	T	C	0.0413
		rs1800469			19	41354391	intron variant	A	G	0.368
Homocysteine synthesis	MTHFR	rs1801133			1	11796321	missense	T	C	0.2454
Antioxidant enzymes	*SOD1*	rs17880135			21	31669690	758 bp 3′ of SOD1	G	T	0.0276
	*SOD1*	rs1041740			21	31667849	intron variant	T	C	0.2428
	*SOD1*	rs202446			21	31656328	intron variant^a^	T	G	0.0755
	*SOD2*	rs4880			6	159692840	missense Val16Ala	G	A	0.4107
	*CAT*	rs1001179			11	34438684	240 bp 5′ of CAT	T	C	0.1256
	*GPX1*	rs1050450	rs17080528	0.98	3	49352409	Downstream gene variant^b^	T	C	0.2175
	*GPX3*	rs870406			5	151021040	intron variant	A	G	0.0974
	*GPX4*	rs713041			19	1106616	synonymous	T	C	0.401
NER	*ERCC2 (XPD)*	rs1799793			19	45364001	Missense Asp312Asn	T	C	0.1945
	*ERCC2*	rs171140			19	45361744	intron variant	C	A	0.367
	*ERCC2*	rs13181			19	45351661	missense 500pb 3′ of ERCC2	G	T	0.2366
	*ERCC4*	rs3136166			16	13938236	intron variant	G	T	0.4249
BER	*OGG1*	rs1052133			3	9757089	missense Ser326Cys	G	C	0.3021
	*MUTYH*	rs3219489			1	45331833	missense Gln338His	G	C	0.3135
	*XRCC1*	rs25487			19	43551574	missense Gln399Arg	T	C	0.2604
Phase-II metabolism	*GSTP1*	rs1695	rs749174	0.92	11	67585782	missense Ile105Val	A	G	0.2438
	*GSTO1*	rs4925	rs2164624	0.93	10	104253687	intron variant^b^	A	G	0.1879
	*GSTO2*	rs156697			10	104279427	missense Asn142Asp	G	A	0.4407
Genes related with mortality in hemolyzed s	*MGP*	rs4236			12	14882147	missense Thr83Ala	C	T	0.3854
	*KL*	rs1207568			13	33016046	22 bp 5′ of KL	A	G	0.1601
*patients*	*KL*	rs577912			13	33036014	intron variant	T	G	0.1953

Chr. chromosome; *according to Haploreg; LD linkage disequilibrium; ^a^http://www.ncbi.nlm.nih.gov/pubmed. ^b^http://www.ensembl.org/index.html; ^c^related to pathological process characteristic of chronic kidney disease.

Table 3 shows the observed associations (*P* < 0.05) between the candidate SNPs and CKD susceptibility in the entire study population. When the analysis was adjusted for age and gender, three SNPs showed an association under the dominant model. These SNPs were rs17080528 in the *GPX1* gene, that encodes one of the most important antioxidant enzymes in humans (OR = 1.87, *P* = 0.001) and rs2164624 and rs156697 in the *GSTO1* and *GSTO2* genes, both involved in the metabolism of xenobiotics and carcinogens (OR = 0.50, *P* = 0.0007, and OR = 0.57, *P* = 0.013, respectively). For the SNPs rs12917707 in *UMOD*, that acts as a constitutive inhibitor of calcium crystallization in renal fluids, and rs4236 in *MGP*, which encodes a protein acting as an inhibitor of bone formation, the associations were identified under an additive inheritance model (allelic OR = 0.72, *P* = 0.043 and allelic OR = 0.75, *P* = 0.023, respectively).

**Table 3:** Positive associations found between candidate SNPs and chronic kidney disease (CKD) susceptibility.

Gene	Without co-variables							Adjusted for age and sex		
	SNP	Geno-type	Affec-ted	Un- affected	OR	95%CI	*P*	OR	95% CI	*P*
*GPX1*	rs17080528	CC	211	89	1.00					
		CT	246	60	1.73	1.19–2.52	**0**.**004**	1.91	1.28–2.84	**0**.**002**
		TT	58	15	1.63	0.88–3.03	0.122	1.71	0.89–3.28	0.106
		T			1.44	1.09–1.91	**0**.**010**	1.52	1.13–2.04	**0**.**005**
		CT + TT	304	75	1.71	1.2–2.44	**0**.**003**	1.87	1.28–2.72	**0**.**001**
*GSTO1*	rs2164624	GG	215	45	1.00					
		GA	241	98	0.51	0.35–0.77	**0**.**001**	0.48	0.31–0.73	**0**.**0005**
		AA	71	25	0.59	0.34–1.04	0.067	0.58	0.32–1.05	0.071
		A			0.72	0.55–0.92	**0**.**010**	0.70	0.53–0.91	**0**.**008**
		GA + AA	312	123	0.53	0.36–0.78	**0**.**001**	0.50	0.33–0.75	**0**.**0007**
*GSTO2*	rs156697	AA	173	35	1.00					
		AG	254	89	0.58	0.37–0.89	**0**.**014**	0.60	0.38–0.96	**0**.**031**
		GG	80	33	0.49	0.28–0.85	**0**.**010**	0.49	0.28–0.87	**0**.**015**
		G			0.69	0.53–0.90	**0**.**006**	0.70	0.53–0.91	**0**.**008**
		AG + GG	334	122	0.55	0.36–0.84	**0**.**006**	0.57	0.37–0.89	**0**.**013**
*UMOD*	rs12917707	GG	335	92	1.00					
		GT	156	50	0.86	0.58–1.27	0.441	0.82	0.54–1.24	0.344
		TT	15	12	0.34	0.16–0.76	**0**.**008**	0.39	0.17–0.90	**0**.**027**
		T			0.71	0.53–0.97	**0**.**031**	0.72	0.52–0.99	**0**.**043**
		GT + TT	171	62	0.76	0.52–1.10	0.142	0.74	0.50–1.09	0.13
*MGP*	rs4236	CC	225	63	1.00					
		CT	179	55	0.91	0.60–1.36	0.658	0.81	0.52–1.25	0.340
		TT	84	37	0.64	0.39–1.02	0.063	0.55	0.33–0.91	**0**.**020**
		T			0.81	0.64–1.03	0.08	0.75	0.58–0.96	**0**.**023**
		CT + TT	263	92	0.80	0.55–1.16	0.234	0.70	0.48–1.04	0.08

Case-control analysis, OR, odds ratio; CI, confidence interval.

### SNPs associated with related pathologies

It is known that patients with CKD have at the same time other diseases, which are related to the presence of renal failure, either as a cause or as a consequence. Among them we can indicate hypertension (HT), cardiovascular disease (CVD), and diabetes mellitus (DM) and, in some cases a medical history of cancer. In our study we observed a high incidence of HT (91.5%), CVD (45.2%), DM (32%), and previous cancer (30%) in patients with CKD.

When the associations between candidate SNPs and pathologies related to CKD were considered, some associations were observed (Table 4). For HT, two genes, *GPX4*, implicated in the protection of cells against oxidative damage, and *CYP11B2*, with the encoded enzyme catalyzing many reactions involved in drug metabolism and synthesis of cholesterol, steroids, and other lipids, showed an association in the dominant model. *ERCC4*, involved in nucleotide excision repair pathway, showed association in the additive model. With regard to previous cancer history, the *ERCC2* gene, also involved in nucleotide excision repair, showed an association under allelic model (rs13181, rs713041) and dominant model (rs1052133). For CVD we observed an association in the dominant model with the *ERCC2* gene.

**Table 4:** Positive associations observed between candidate SNPs and pathologies related to chronic kidney disease (CKD), case-only analysis.

Pathology	Gene	SNP	Genotype	No pathology^§^	With pathology^$^	OR^#^	95% CI^#^	*P*^*#*^
Hypertension	***GPX4***	rs713041*	TT	8	190	1.00		
			TC	14	128	0.39	0.16–0.96	**0**.**040**
			CC	15	85	0.24	0.10–0.58	**0**.**002**
			C			0.50	0.32–0.76	**0**.**001**
			TC + CC	29	213	0.31	0.14–0.70	**0**.**005**
	***CYP11B2***	rs1799998	AA	22	138	1.00		
			AG	18	228	2.01	1.04–3.89	**0**.**039**
			GG	5	105	3.63	1.23–9.20	**0**.**018**
			G			1.89	1.19–3.00	**0**.**007**
			AG + GG	23	333	2.30	1.24–4.28	**0**.**008**
	***ERCC4***	rs3136166	TT	13	200	1.00		
			TG	21	220	0.67	0.33–1.38	0.281
			GG	10	46	0.29	0.12–0.71	**0**.**007**
			G			0.55	0.35–0.88	**0**.**011**
			TG + GG	31	266	0.55	0.28–1.08	0.083
Previous cancer	***ERCC2***	rs13181	TT	114	61	1.00		
			TG	124	55	0.84	0.53–1.32	0.444
			GG	39	6	0.29	0.11–0.72	**0**.**008**
			G			0.66	0.47–0.92	**0**.**016**
			TG + GG	163	61	0.70	0.45–1.09	0.116
	***ERCC2***	rs713041*	TT	104	40	1.00		
			TC	83	34	1.17	0.67–2.03	0.584
			CC	48	33	1.87	1.03–3.37	**0**.**038**
			C			1.35	1.01–1.82	**0**.**046**
			TC + CC	131	67	1.43	0.88–2.32	0.146
	***ERCC2***	rs1052133	CC	175	65	1.00		
			CG	87	54	1.73	1.10–2.72	**0**.**019**
			GG	16	3	0.63	0.17–2.28	0.481
			G			1.29	0.89–1.87	0.179
			CG + GG	103	57	0.58	1.01–2.46	**0**.**044**
Cardiovascular disease	***ERCC2***	rs1799793	TT	144	107	1.00		
			TC	90	109	1.71	1.15–2.54	**0**.**008**
			CC	26	23	1.16	0.61–2.21	0.644
			C			1.28	0.96–1.69	0.088
			TC + CC	116	132	1.58	1.09–2.29	**0**.**016**

Case-case analysis, OR, odds ratio; CI, confidence interval.

^#^ORs and the corresponding 95% CIs were adjusted for age and gender.

^$^The number of genotypes may differ due to missing clinical data and/or genotypes; overall the genotype call rate was over 0.92.

*Genotype call rate for rs713041 was 0.81; both among individuals with and without the pathology.

### SNPs associated with clinical/biochemical parameters

CKD patients are characterized by a defined biochemical profile acting as a clinical indicator. To detect associations between the selected SNPs and clinical parameters the main analyses were done in a combined case-control population, using both logistic and linear regression models with the median or normal value as a cut-off, considering normal values according to the international system. They are used by the Puigvert Foundation, as standard protocols, and can be seen in Supplementary Table S1 for each of the selected clinical parameters. Case-only analysis to verify the associations was done using only linear regression model. The obtained results are shown in Table S1. As indicated, nine biochemical parameters showed any kind of statistical association with defined genes: creatinine, glomerular filtration rate, hemoglobin, erythropoietin resistance index, albumin, phosphorus, parathyroid hormone, C-reactive protein, and ferritin.

Genetic variants codifying for antioxidant enzymes were associated with the levels of creatinine, glomerular filtration rate, erythropoietin resistance index, albumin and phosphorus levels, C-reactive protein, and ferritin levels (rs17080528 from *GPX1* gene, rs713041 from *GPX4*, rs4880 from *SOD2* gene, rs17880135, rs202446, rs1041740 from *SOD1* gene). Other genetic variants coding for phase II metabolism enzymes, were also associated with the levels of creatinine, GFR, albumin, C-reactive protein and ferritin (rs2164624 from *GSTO1* gene, rs156697 from *GSTO2* gene, rs749174 from *GSTP1* gene). Genetic variants of genes involved in DNA repair were strongly associated with hemoglobin and albumin levels (rs171140 from *ERCC2* gene) and showed borderline associations with the levels of the C-reactive protein (rs25487 for *XRCC1*) and phosphorus (rs3136166 from *ERCC4* gene). Other variants associated with renal pathology itself showed a significant relationship with the levels of creatinine, GFR, and hemoglobin, as well as RIE, PTH and C-reactive protein (rs577912, rs1207568 from *KL* gene, rs4236 from *MGP* gene, rs17319721 from *SHROOM3* gene, rs881858 from *VEGFA* gene, rs12917707 from *UMOD* gene, and rs12460876 from *SLC7A9* gene).

Some genes related to the immune response showed a moderate association with the GFR, phosphorus and PTH levels (rs5498 from the *ICAM-1* gene, rs2070874 from *IL-4* gene, rs17561 from *IL-1A* gene, and rs1800797 from the *IL-6* gene). In addition, a variant in the gene *MTHFR*, related to the homocysteine synthesis, showed a moderate association with the RIE.

Replication in the case-only setting supported the associations of the combined case-control analysis, with reduced significance for parameters, for which the cases and controls showed clearly distinct patterns, such as creatinine and glomerular filtration rate. For those parameters, a few additional associations appeared, however, with genes among the same group of antioxidant enzymes (*SOD1* rs1041740) and renal pathology (*KL* rs1204568, *VEGFA* rs881858) as in the combined analysis. Also for hemoglobin, two additional variants belonging to the DNA repair pathway emerged (*ERCC2* rs1799793, *ERCC4* rs3136166). For other parameters, such as erythropoietin resistance index, albumin, phosphorus, parathyroid hormone, C-reactive protein and ferritin, the associations were similar, or even stronger than in the combined analysis, due to the fact that clinical data were available mostly for cases.

## Discussion

This study succeeded to demonstrate associations between five SNPs and CKD in the list of 38 SNPs selected in the 31 candidate genes. Genes showing associations were *GPX1*, *GSTO1*, *GSTO2*, *UMOD*, and *MGP*.

*GPX1* is the major isoform of *GPX* that is expressed in the normal kidney; this accounts for 96% of kidney GPX activity and shows a protective role against oxidative stress^26^. Pro198Leu and Pro197Leu variants (strongly associated with our variant, LD r^2^ = 0.98) have been reported to be associated with reduction of GPX1 activity^27^, and it has been suggested that *GPX1* is a possible candidate gene for CVD risk^28^ that, as previously indicated, is a pathology strongly linked to CKD.

Glutathione S-transferases (GSTs) are detoxification enzymes playing an important role in the conjugation of endogenous or exogenous xenobiotic toxins to glutathione (GSH). The family of cytosolic GSTs has different classes, including the Omega (GSTO) class^29^. Polymorphisms in *GSTO1* and *GSTO2*, members of the Omega class, might influence the level of oxidative stress^30^. *GSTO1* (rs4925) and *GSTO2* (rs156697) genotypes have been associated with worse prognosis and shorter survival in bladder cancer patients^31^.

The *UMOD* gene encodes for uromodulin protein acting as a constitutive inhibitor of calcium crystallization in renal fluids^32^. The SNP rs12917707 was found to be associated with both glomerular filtration rate and better kidney function in two GWASs^33,34^. Seven SNPs of the *UMOD* gene that are in high LD with rs12917707 were also associated with CKD at a genome-wide significant level^35^ and, in general, many studies independently corroborate earlier evidence for the association between *UMOD* and CKD^36^.

The *MGP* gene encodes a protein acting as an inhibitor of bone formation. The rs4236 variant results in a missense mutation influencing the calcification process and affecting atherosclerotic plaques^37^. It is also known that the variant form is associated with a decreased quantity of coronary artery calcification^38^. In this context, our results are consistent with those reported in the literature showing a protective effect with respect to CKD^39^. A recent publication on the Spanish Nefrona Cohort also found the association of the rs4236 SNP of the *MGP* gene with CKD^40^.

Different pathologies like hypertension, cardiovascular disease and cancer are strongly linked with CKD. Although in our study none of the genes associated with CKD were associated with these pathologies, positive associations with other candidate SNPs and genes were observed. *GPX4*, *CYP11B2*, and *ERCC4* genes were associated with hypertension. The phospholipid hydroperoxide GPX (GPX4) is a common intracellular selenoprotein that reduces lipid hydroperoxides and regulates leukotriene biosynthesis and cytokine signaling pathways. The SNP rs713041 causes a C to T substitution in a region of the *GPX4* gene corresponding to the 3´-untranslated region of the messenger RNA altering protein binding^41^. Although no association was observed in a Japanese CKD population^42^, a direct relationship between the rate of change of plasma GPX activity and the rate of change of glomerular filtration index was observed^43^. *The CYP11B2* gene, which encodes the human aldosterone synthase, is a cytochrome P450 enzyme that catalyzes the terminal steps of aldosterone synthesis in the zona glomerulosa cells of the adrenal cortex^44^. The rs1799998 polymorphism has been suggested to be associated with genetic predisposition to cardiovascular diseases, such as myocardial infarction and hypertension^45^. Our study agrees with those researches revealing an association with increased risk of hypertension among CKD patients^46^. Finally, *ERCC4* is involved in nucleotide excision repair (NER) pathway, with a reported association with cancer^47^. This is a new finding, as no previous report found associations between this SNP and kidney diseases or hypertension.

With regard to previous cancer history and cardiovascular disease, an association with *ERCC2* was observed. *ERCC2* is an important DNA repair gene in the NER pathway that has been associated with cancer incidence^48^, and with cardiovascular disease^49^ but no previous reports linked this gene with kidney failure in humans. Nevertheless, *ERCC* has shown to be associated with age-related vascular dysfunction in a mouse model^50^. Interestingly in our study, this gene was associated with both CVD and with a previous cancer history among CKD patients.

Since CKD is characterized by changes in clinical parameters, which in our case are continuous values, both a linear and a logistic regression model, with either the median or the normal value as a cut-off were carried out. Several associations between clinical parameters and selected SNPs were observed as indicated in Table S1. All genes showing association with CKD showed also at least one association with the evaluated clinical parameters. *GPX1*, *GSTO*, *KL*, *and MGP* genes showed associations with the creatinine levels and with the glomerular filtration rate, which are strongly linked to CKD. In addition, *GPX1* and *GPX4* genes were also associated with phosphorus levels, *GPX4* with ferritin levels, *GSTO* with albumin levels, and *KL* with the resistance index to erythropoietin. The *UMOD* gene was associated with the levels of parathyroid hormone.

In consonance with the large importance of cytokines in inflammatory diseases and considering that inflammation is closely related to mineral disorders in CKD^51,52^ we found an association between *IL-4* gene and phosphorus levels. We also identified an association between *IL1A*, *IL-6*, and *ICAM-1* genes and parathyroid hormone (PTH) values; and *ICAM-1* gene also with the glomerular filtration rate. Previous investigations demonstrated the effect of *IL-6*, *IL-4*, and *ICAM* polymorphisms in end-stage renal disease patients^53^. Interestingly, both cytokines (IL1A and IL-6) have been implicated as key factors linking malnutrition, accelerated atherogenesis, and excessive morbidity and mortality in end-stage renal disease (ESRD) patients in hemodialysis^54^. In addition, *IL-4* was also associated with phosphorus levels. The same polymorphisms we evaluated for *IL-4* and *IL-6* genes were also associated with kidney function and CKD prevalence in a large Japanese population^55^ where *IL-4* was found to be associated with glomerulonephritis^56^, and increased levels of phosphorus as well as parathyroid cell proliferation^57^, that would support our findings. Surprisingly, there was a lack of association among SNPs of *IL-1* and *IL-6* and CRP, giving the well-known relationship between CRP and inflammation. It would be interesting to analyze the possible association of these SNPs with the neutrophil-lymphocyte and platelet-lymphocyte ratio, which are considered prognostic markers associated with inflammation in many diseases including CKD, but unfortunately this information was not available.

With regard to the selected antioxidant genes, in addition of the role of *GPX*, *SOD* genes were also associated with biomarkers such as erythropoietin resistance index, albumin and phosphorus levels, C-reactive protein and ferritin levels. These associations agree with a previous study^58^, supporting the role of oxidative stress in the progression of several diseases, including CKD. Our findings also agree with previous researches showing that *SOD* was associated with advanced nephropathy^59^.

Three of the genes involved in DNA repair, *ERCC2*, *ERCC4*, and *XRCC1* were associated with different clinical parameters. *ERCC2* was associated with albumin and hemoglobin levels, *ERCC4* with phosphorus levels, while *XRCC1* was associated with C-reactive protein values. *ERCC* genes are involved in DNA repair, in particular in the nucleotide excision repair pathway, and different SNPs have been associated with kidney pathologies such as renal cell carcinoma^60^. In fact, *ERCC1* and *ERCC4* play important roles in the development of nephropathies, as demonstrated in mammalian models^61^. The variant rs25487 of the *XRCC1* gene confers increased risk for the development of ESRD^62,63^.

In our study, the *GSTP1* gene was associated with C-reactive protein and ferritin levels. This gene is involved in a wide range of detoxification reactions that protect cells from carcinogens^64^. GSTs provide protection against reactive oxygen species and the electrophilic metabolites of carcinogens. Interestingly the role of *GSTP1* (together with *GSTA1*, *GSTM1*, and *GSTT)* genotypes was already determined in a group of end-stage renal disease patients showing that those individuals carrying the null alleles showed increased susceptibility towards oxidative and carbonyl stress^65^. The Klotho (*KL*) gene encodes the klotho protein controlling multiple ion channels and growth factor signaling pathways, including insulin, IGF-1, and Wnt signaling.

The Klotho gene was associated with high creatinine levels, glomerular filtration rate, and erythropoietin resistance index. *KL* expression in kidneys was reduced in patients with chronic renal failure^66^, which would imply that the reduction of KL protein may be relevant in the pathophysiology of kidney disease. Our results would agree with those indicating a role in the increased risk observed in different pathologies associated with CKD^67^.

*SHROOM3*, *VEGFA*, *UMOD*, and *SLC7A9*, were associated with hemoglobin and parathyroid hormone levels, with erythropoietin resistance index, with parathyroid hormone levels, and with ferritin values, respectively. Interestingly these four genes were previously associated with CKD in GWAS studies^23,68–70^. *SHROOM3* encodes an actin-binding protein expressed in the kidney, where it may have an important role in the morphogenesis of epithelial tissues during development^71^. *VEGFA* encodes vascular endothelial growth factor A, and some variants have been identified related to nephrogenesis^23^. *UMOD* encodes uromodulin which is the most abundant protein in normal urine^72^ having antimicrobial properties providing defense against uropathogens responsible for urinary tract infections; in addition, it may also play a role in preventing crystallization of calcium and uric acid in kidneys and urine^73^. The *SLC7A9* gene encodes the neutral and basic amino acid transport protein (rBAT) involved in the transport of the urinary dibasic amino acids across the renal tubular membrane^74^. In spite of their importance in kidney physiology, we did not find association between these genes and CKD; nevertheless, we were able to detect their modulatory role on some of the biochemical parameters that are characteristics of CKD patients. A possible explanation for the lack of association of these SNPs with CKD could be the small sample size of the control group.

MTHFR, a folate-dependent enzyme, plays an important role in the conversion of homocysteine to methionine, being important for most of the biological processes. The variant rs1801133 of the *MTHFR* gene is relatively common and it has been studied for a long time. There is a wide list of disorders in different populations around the world affected by this SNP, and *MTHFR* variants are associated with susceptibility of type 2 diabetes mellitus in diabetic nephropathy^75^. Our results showed an association between the *MTHFR* variant and the resistance index to the erythropoietin. Different studies show that ESRD patients homozygous for the mutant allele rs1801133 have increased mortality risk^76^, and associations of the *MTHFR* gene with CKD progression have also been reported^77,78^.

A detailed discussion of the pros and cons of SNP association studies in the clinical context of CKD is outside the scope of this article, and a large number of comparisons tested may hinder the clarity of the results. Many of these studies rely on small samples, often being limited by the logistics of clinical study designs. Therefore, the ratio of the number of variables to the number of individuals/observations grows even higher, placing additional constraints on the analysis methods. An additional difficulty here lies in incorporating different data types (e.g., SNPs from different kinds of genes and metabolite measurements from metabolomics studies) into the same analysis framework, which is something that the traditional analysis using parametric statistical methods are not particularly efficient at either. Thus, small numbers of patients in some analysis, multiple variables analyzed, with possible correlation between them, are perhaps the most difficult challenge, required in this study, to associate complex variants with the CKD.

The overall conclusion of this study is that variants in *GPX1*, *GSTO1*, *GSTO2*, *UMOD*, and *MGP* genes are associated with CKD. In addition, other genes were found to be associated with CKD related pathologies, such as hypertension (*GPX4*, *CYP11B2*, *ERCC4*), cancer predisposition (*ERCC2*), and cardiovascular disease (*ERCC2*). Finally, associations with classical CKD biochemical parameters were found for creatinine (*GPX1*, *GSTO1*, *GSTO2*, *KL*, *MGP*), glomerular filtration rate (*GPX1*, *GSTO1*, *KL*, *ICAM-1*, *MGP*), hemoglobin (*ERCC2*, *SHROOM3*), resistance index erythropoietin (*SOD2*, *VEGFA*, *MTHFR*, *KL*), albumin (*SOD1*, *SOD2*, *GSTO2*, *ERCC2*), phosphorus (*IL-4*, *ERCC4*, *SOD1*, *GPX1*, *GPX4*) parathyroid hormone (*IL-1A*, *IL6*, *SHROOM3*, *UMOD*, *ICAM-1*), C-reactive protein (*SOD2*, *GSTP1*, *XRCC1*), and ferritin (*SOD2*, *GSTP1*, *SLC7A9*, *GPX4*).

## Methods

### Ethics statement

All individuals participating in the study provided written informed consent, and blood samples were collected under protocols approved by the Ethics Committee of the Puigvert Foundation from Barcelona and Josep Trueta Hospital from Girona, in accordance with the tenets of the Declaration of Helsinki. In addition to the genotyping studies, peripheral blood samples were also used to determine standard biochemical parameters relevant for CKD.

### Study populations

The study involved a total of 722 European-Spanish adults, including 548 patients suffering kidney pathologies at different stages, and 174 controls. All patients had a reduced glomerular filtration rate (GFR < 60 mL/min/1.73 m^2^). In total, we had 338 men and 210 women (62% and 38%, respectively) as CKD patients. Healthy controls were 105 men and 67 women (61% and 39%, respectively).

General characteristics of all patients are shown in Table 1. In addition to the 133 patients recruited from the hospital J. Trueta (Girona), 415 patients and all controls were randomly recruited at the Puigvert Foundation, Barcelona, over a period of 7 years. Controls were selected from the urology clinic outpatients suffering from either prostatic pathology, urinary tract infections or kidney stones, and all had normal GFR, according to their ages. All controls and 415 patients belong to our previous work^19^.

### Gene and SNP selection, and genotyping

*Gene and SNP selection*. A total of 38 SNPs from 31 candidate genes were selected. Some of them were previously reported in a GWAS to be associated with CKD (GLO1, *SLC7A9*, *SHROOM3*, *UMOD*, *and VEGFA*)^23,33,35,79^, other were related to pathological processes characteristic of CKD, such as cytokines (*IL-1A*, *IL-4*, *IL-6*, *IL10*, *TNF-a* and *ICAM-1*), renin-angiotensin-aldosterone system (*AGT* and *CYP11B2*), proteins involved in fibrogenesis (*TGFB1*), and in homocysteine synthesis (*MTHFR*). Some genes coding for antioxidant enzymes were also included (*SOD1*, *SOD2*, *CAT*, *GPX1*, *GPX3*, and *GPX4*). Moreover, genes involved in DNA repair pathways such as nucleotide excision repair (NER) genes (*ERCC2*, and *ERCC4*) and base excision repair (BER) genes (*OGG1*, *MUTYH*, *XRCC1)*, and phase-II metabolism (*GSTP1*, *GSTO1*, and *GSTO2*) were included. Finally, other genes related to mortality in hemodialysis patients, vascular calcification and aging (*KL* and *MGP*)^80,81^ were also incorporated into the study. The gene and SNP selection were based on published studies reporting associations of SNPs with CKD, or related phenotypes, and all selected SNPs had a minor allele frequency (MAF) > 10%. Table 2 shows details of the SNPs studied in our population. When no genotyping assay was available for the selected SNP another SNP in high linkage disequilibrium (r^2^ > 0.8) was genotyped instead (alternative SNPs in Table 2).

Genotyping was carried out using the TaqMan SNP genotyping assays (Life Technologies), according to the manufacturer’s guidelines. To assure the genotyping reliability, repeated analysis was performed in a randomly selected 10% of samples (quality controls); no discrepancies between the genotypes were observed. KASP allelic discrimination method (LGCgenomics, Middelsex, UK) was used to genotype the SNPs rs1800896, rs1800470, rs1799793, and rs1207568. DNA amplification was performed according to the LGC genomics’ PCR conditions. Genotype detection for all SNPs was performed using a ViiA™ 7 v1.2.1 (Applied Biosystems) and allelic discrimination was performed with 95% confidence. Further information can be found in the PhD of the first author^82^.

### Statistical analysis

For the comparison of means of the different clinical parameters, between cases and controls, the Mann Whitney test was used. For the analysis of the pathologies associated with CKD, the Fisher test was performed.

In the association study, samples with <50% call rate were excluded. The observed genotype frequencies in controls were tested for Hardy-Weinberg equilibrium using the Chi-square test. Odds ratios (ORs) and 95% confidence intervals (95% CIs) for associations between genotypes and CKD, associated phenotypes and clinical parameters converted to binary variables were estimated by logistic regression while linear regression was used for continuous variables. The analyses were done considering two models, one without adjustment and a second adjusting for age and gender. Statistical significance was determined by a *P*-value lower than 0.05. The analyses were performed using the following statistical software: the Statistic Package of Social Sciences (SPSS) software for Windows version 19.0, PLINK 1.90, https://www.cog-genomics.org/plink2 ^83^ and Rx64 3.1.3 for^82^ Windows, http://www.r-project.org.

## Supplementary information

Supplementary Information.
