# Supplementary material for: Genetic Variants Associated with Chronic Kidney Disease in a Spanish Population
Source: Sci Rep. 2020 Jan 10;10:144. doi: 10.1038/s41598-019-56695-2 (PMC6954113; doi:10.1038/s41598-019-56695-2)
Supplement: Supplementary file 1 — Supplementary Information. [file 41598_2019_56695_MOESM1_ESM.docx]

Genetic Variants Associated with Chronic Kidney Disease in a Spanish Population

Zuray Corredor^1,+^, Miguel Inácio da Silva Filho^2,+^, Lara Rodríguez-Ribera^1^, Antonia Velázquez^1,3^, Alba Hernández^1,3^ Calogerina Catalano^2^, Kari Hemminki^2^, Elisabeth Coll^4^, Irene Silva^4^, Juan Manuel Diaz^4^, José Ballarin^4^, Martí Vallés Prats^5^, Jordi Calabia Martínez^5^, Asta Försti^2,+^, Ricard Marcos^1,3,+,^* and Susana Pastor^1,3,+,^*

**SUPPLEMENTARY TABLE**

**Supplementary Table**

**Table S1.** Positive associations observed between candidates SNPs and different clinical parameters related to chronic kidney disease (CKD). Available data from both cases and controls were used for the main analyses by logistic and linear regression. Case-only analysis was done only by linear regression.

| **Clinical**  **Parameters**  **(normal values)**  (Cut-off value) | **Gene** | **SNP** | **Genotype** | **Group1** | **Group 2** | **OR^#^** | **95%CI^#^** | ***P_logistic_*** | | ***P_linear_*** | ***P_linear_***  ***_cases only_*** |
| --- | --- | --- | --- | --- | --- | --- | --- | --- | --- | --- | --- |
| **Creatinine**  **(45-80 µmol/L)**  (100 µmol/L*) | ***GPX1*** | rs17080528 | CC | 88 | 74 | 1.00 |  | |  |  |  |
|  |  |  | CT | 62 | 88 | 1.73 | 1.07-2.78 | | **0.025** | **0.002** | **0.09** |
|  |  |  | TT | 16 | 13 | 0.85 | 0.37-1.96 | | 0.702 | 0.725 | 0.72 |
|  |  |  | T |  |  | 1.20 | 0.84-1.71 | | 0.314 | 0.142 | 0.47 |
|  |  |  | CT+TT | 77 | 103 | 1.53 | 0.98-2.41 | | 0.063 | **0.010** | 0.16 |
|  | ***GSTO1*** | rs2164624 | GG | 46 | 79 | 1.00 |  | |  |  |  |
|  |  |  | GA | 97 | 76 | 0.46 | 0.28-0.76 | | **0.002** | **0.0008** | 0.29 |
|  |  |  | AA | 27 | 25 | 0.54 | 0.27-1.07 | | 0.078 | 0.107 | 0.56 |
|  |  |  | A |  |  | 0.66 | 0.48-0.92 | | **0.015** | **0.016** | 0.39 |
|  |  |  | GA+AA | 124 | 101 | 0.48 | 0.3-0.77 | | **0.002** | **0.0006** | 0.28 |
|  | ***GST02*** | rs156697 | AA | 37 | 61 | 1.00 |  | |  |  |  |
|  |  |  | AG | 87 | 87 | 0.73 | 0.43-1.26 | | 0.26 | 0.121 | 0.76 |
|  |  |  | GG | 35 | 28 | 0.50 | 0.25-0.97 | | **0.041** | 0.149 | 0.98 |
|  |  |  | G |  |  | 0.71 | 0.51-0.99 | | **0.041** | 0.117 | 0.91 |
|  |  |  | AG+GG | 122 | 115 | 0.65 | 0.39-1.09 | | 0.103 | **0.0006** | 0.80 |
|  | ***KL*** | rs577912 | GG | 117 | 114 | 1.00 |  | |  |  |  |
|  |  |  | GT | 45 | 50 | 1.13 | 0.68-1.87 | | 0.642 | 0.110 | 0.09 |
|  |  |  | TT | 1 | 8 | 12.06 | 1.37-106.4 | | **0.025** | **0.009** | 0.39 |
|  |  |  | T |  |  | 1.47 | 0.95-2.29 | | 0.084 | **0.008** | 0.08 |
|  |  |  | GT+TT | 46 | 58 | 1.31 | 0.80-2.15 | | 0.279 | **0.031** | 0.07 |
|  | ***MGP*** | rs4236 | GG | 65 | 85 | 1.00 |  | |  |  |  |
|  |  |  | GA | 56 | 52 | 0.61 | 0.36-1.03 | | 0.062 | 0.231 | 0.78 |
|  |  |  | AA | 36 | 33 | 0.57 | 0.31-1.05 | | 0.073 | **0.047** | 0.49 |
|  |  |  | A |  |  | 0.74 | 0.55-0.99 | | **0.043** | **0.041** | 0.61 |
|  |  |  | GA+AA | 92 | 85 | 0.59 | 0.37-0.95 | | **0.028** | **0.066** | 0.88 |
|  |  |  |  |  |  |  |  | |  |  |  |
|  | *SOD1* | rs1041740 | CC | 69 | 73 | 1.00 |  | |  |  |  |
|  |  |  | CT | 79 | 74 | 0.87 | 0.53-1.41 | | 0.56 | 0.61 | **0.021** |
|  |  |  | TT | 19 | 23 | 1.13 | 0.55-2.36 | | 0.76 | 0.47 | 0.50 |
|  |  |  | T |  |  | 1.00 | 0.72-1.40 | | 0.99 | 0.44 | 0.16 |
|  |  |  | CT+TT | 98 | 97 | 0.92 | 0.58-1.45 | | 0.71 | 0.51 | **0.035** |
| **Glomerular filtration**  **rate**  **(>60mL/min/**  **1.75m^2^)** (60mL/min/  1.75m^2^)** | ***GPX1*** | rs17080528 | CC | 93 | 69 | 1.00 |  | |  |  |  |
|  |  |  | CT | 60 | 90 | 2.17 | 1.33-3.54 | | **0.002** | **0.002** | 0.09 |
|  |  |  | TT | 15 | 14 | 1.15 | 0.49-2.67 | | 0.748 | 0.947 | 0.99 |
|  |  |  | T |  |  | 1.44 | 1.00-2.08 | | **0.048** | 0.088 | 0.33 |
|  |  |  | CT+TT | 75 | 104 | 1.95 | 1.23-3.10 | | **0.005** | **0.006** | 0.13 |
|  | ***GSTO1*** | rs2164624 | GG | 49 | 76 | 1.00 |  | |  |  |  |
|  |  |  | GA | 98 | 75 | 0.49 | 0.29-0.80 | | **0.005** | **0.0005** | 0.44 |
|  |  |  | AA | 25 | 27 | 0.70 | 0.35-1.42 | | 0.325 | 0.114 | 0.48 |
|  |  |  | A |  |  | 0.74 | 0.53-1.04 | | 0.082 | **0.016** | 0.39 |
|  |  |  | GA+AA | 123 | 102 | 0.53 | 0.33-0.85 | | **0.009** | **0.0008** | 0.38 |
|  | ***KL*** | rs577912 | GG | 117 | 114 | 1.00 |  | |  |  |  |
|  |  |  | GT | 47 | 48 | 1.04 | 0.62-1.74 | | 0.874 | 0.193 | 0.10 |
|  |  |  | TT | 1 | 8 | 13.44 | 1.48-122.4 | | **0.021** | **0.007** | 0.40 |
|  |  |  | T |  |  | 1.40 | 0.90-2.20 | | 0.136 | **0.014** | 0.09 |
|  |  |  | GT+TT | 68 | 56 | 1.22 | 0.74-2.01 | | 0.428 | 0.058 | 0.08 |
|  | ***ICAM-1*** | rs5498 | AA | 46 | 34 | 1.00 |  | |  |  |  |
|  |  |  | AG | 75 | 91 | 1.89 | 1.06-3.37 | | **0.031** | **0.022** | 0.81 |
|  |  |  | GG | 49 | 48 | 1.28 | 0.67-2.41 | | 0.456 | 0.312 | 0.99 |
|  |  |  | G |  |  | 1.11 | 0.81-1.52 | | 0.532 | 0.385 | 0.95 |
|  |  |  | AG+GG | 124 | 139 | 1.63 | 0.95-2.80 | | 0.075 | **0.048** | 0.88 |
|  | ***MGP*** | rs4236 | GG | 66 | 84 | 1.00 |  | |  |  |  |
|  |  |  | GA | 57 | 51 | 0.57 | 0.34-0.98 | | **0.044** | 0.107 | 0.92 |
|  |  |  | AA | 36 | 33 | 0.57 | 0.31-1.06 | | 0.076 | 0.074 | 1.00 |
|  |  |  | A |  |  | 0.73 | 0.54-0.99 | | **0.042** | **0.049** | 0.98 |
|  |  |  | GA+AA | 93 | 84 | 0.57 | 0.36-0.92 | | **0.022** | **0.042** | 0.94 |
|  |  |  |  |  |  |  |  | |  |  |  |
|  | ***SOD1*** | rs1041740 | CC | 70 | 72 | 1.00 |  | |  |  |  |
|  |  |  | CT | 81 | 72 | 0.81 | 0.50-1.33 | | 0.41 | 0.64 | **0.019** |
|  |  |  | TT | 18 | 24 | 1.23 | 0.58-2.58 | | 0.59 | 0.51 | 0.50 |
|  |  |  | T |  |  | 1.01 | 0.72-1.42 | | 0.95 | 0.76 | 0.16 |
|  |  |  | CT+TT | 99 | 96 | 0.89 | 0.56-1.41 | | 0.61 | 0.86 | **0.018** |
|  |  |  |  |  |  |  |  | |  |  |  |
|  | ***KL*** | rs1207568 | GG | 116 | 129 |  |  | |  |  |  |
|  |  |  | AG | 47 | 37 | 0.69 | 0.40-1.17 | | 0.16 | 0.08 | **0.042** |
|  |  |  | AA | 2 | 7 | 1.70 | 0.33-8.83 | | 0.53 | 0.65 | 0.71 |
|  |  |  | A |  |  | 0.84 | 0.54-1.31 | | 0.44 | 0.25 | 0.10 |
|  |  |  | AG+AA | 49 | 44 | 0.74 | 0.44-1.24 | | 0.25 | 0.13 | **0.048** |
|  | ***VEGFA*** | rs881858 | AA | 69 | 72 | 1.00 |  | |  |  |  |
|  |  |  | AG | 84 | 85 | 1.20 | 0.74-1.95 | | 0.46 | 0.82 | **0.019** |
|  |  |  | GG | 15 | 14 | 1.15 | 0.49-2.70 | | 0.76 | 0.90 | 0.40 |
|  |  |  | G |  |  | 1.12 | 0.78-1.62 | | 0.54 | 0.83 | 0.06 |
|  |  |  | AG+GG | 99 | 99 | 1.19 | 0.75-1.91 | | 0.46 | 0.81 | **0.021** |
|  |  |  |  |  |  |  |  | |  |  |  |
|  | ***GPX4*** | rs713041 | TT | 63 | 76 | 1.00 |  | |  |  |  |
|  |  |  | CT | 35 | 30 | 0.61 | 0.32-1.15 | | 0.13 | 0.24 | 0.80 |
|  |  |  | CC | 40 | 39 | 0.75 | 0.41-1.37 | | 0.35 | 0.09 | **0.024** |
|  |  |  | C |  |  | 0.85 | 0.63-1.14 | | 0.28 | 0.08 | **0.030** |
|  |  |  | CT+CC | 75 | 69 | 0.68 | 0.41-1.14 | | 0.14 | 0.08 | 0.68 |
|  |  |  |  |  |  |  |  | |  |  |  |
|  |  |  |  |  |  |  |  | |  |  |  |
| Hemoglobin  (120-160 g/L)  (130 g/L)* | ***ERCC2*** | rs171140 | CC | 98 | 65 | 1.00 |  | |  |  |  |
|  |  |  | CA | 142 | 153 | 1.74 | 1.17-2.58 | | **0.006** | **0.003** | **0.002** |
|  |  |  | AA | 69 | 72 | 1.57 | 0.98-2.49 | | 0.058 | **0.03** | **0.021** |
|  |  |  | A |  |  | 1.26 | 1.00-1.59 | | **0.047** | **0.025** | **0.016** |
|  |  |  | CA+AA | 211 | 225 | 1.68 | 1.16-2.44 | | **0.006** | **0.002** | **0.002** |
|  | ***SHROOM3*** | rs17319721 | GG | 133 | 99 | 1.00 |  | |  |  |  |
|  |  |  | GA | 132 | 148 | 1.46 | 1.03-2.09 | | **0.036** | 0.302 | 0.96 |
|  |  |  | AA | 44 | 43 | 1.34 | 0.81-2.17 | | 0.250 | 0.891 | 0.91 |
|  |  |  | A |  |  | 1.22 | 0.97-1.55 | | 0.095 | 0.745 | 0.91 |
|  |  |  | GA+AA | 176 | 191 | 1.43 | 1.02-2.01 | | **0.036** | 0.593 | 0.94 |
|  | *GPX4* | rs713041 | TT | 122 | 106 | 1.00 |  | |  |  |  |
|  |  |  | CT | 70 | 93 | 1.51 | 1.00-2.27 | | **0.05** | **0.03** | **0.018** |
|  |  |  | CC | 69 | 57 | 0.96 | 0.62-1.5 | | 0.86 | 0.58 | 0.15 |
|  |  |  | C |  |  | 1.02 | 0.82-1.27 | | 0.85 | 0.37 | 0.41 |
|  |  |  | CT+CC | 139 | 150 | 1.24 | 0.87-1.76 | | 0.24 | 0.09 | **0.019** |
|  | *ERCC2* | rs1799793 | TT | 156 | 134 | 1.00 |  | |  |  |  |
|  |  |  | CT | 117 | 123 | 1.26 | 0.89-1.78 | | 0.20 | 0.08 | **0.027** |
|  |  |  | CC | 29 | 31 | 1.25 | 0.71-2.21 | | 0.43 | 0.51 | 0.29 |
|  |  |  | C |  |  | 1.17 | 0.91-1.50 | | 0.22 | 0.17 | 0.056 |
|  |  |  | CT+CC | 146 | 154 | 1.26 | 0.90-1.75 | | 0.18 | 0.09 | **0.024** |
|  | *ERCC4* | rs3136166 | TT | 118 | 129 | 1.00 |  | |  |  |  |
|  |  |  | GT | 151 | 129 | 0.76 | 0.53-1.08 | | 0.12 | **0.05** | **0.05** |
|  |  |  | GG | 38 | 33 | 0.76 | 0.44-1.30 | | 0.31 | 0.18 | 0.20 |
|  |  |  | G |  |  | 0.83 | 0.65-1.07 | | 0.15 | 0.06 | 0.06 |
|  |  |  | GT+GG | 189 | 162 | 0.76 | 0.54-1.06 | | 0.10 | **0.038** | **0.039** |
| **Resistance index erythropoietin (<10)**  (10)** | ***SOD2*** | rs4880 | AA | 33 | 52 | 1.00 |  | |  |  |  |
|  |  |  | AG | 43 | 55 | 0.81 | 0.45-1.47 | | 0.496 | **0.047** | **0.047** |
|  |  |  | GG | 23 | 30 | 0.82 | 0.41-1.65 | | 0.573 | 0.103 | 0.13 |
|  |  |  | G |  |  | 0.90 | 0.63-1.26 | | 0.530 | 0.068 | 0.08 |
|  |  |  | AG+GG | 66 | 85 | 0.82 | 0.47-1.40 | | 0.461 | **0.032** | **0.036** |
|  | ***VEGFA*** | rs881858 | AA | 35 | 69 | 1.00 |  | |  |  |  |
|  |  |  | AG | 55 | 53 | 0.49 | 0.28-0.85 | | **0.011** | **0.027** | **0.025** |
|  |  |  | GG | 8 | 14 | 0.91 | 0.35-2.37 | | 0.842 | 0.501 | 0.50 |
|  |  |  | G |  |  | 0.73 | 0.49-1.11 | | 0.140 | 0.101 | 0.10 |
|  |  |  | AG+GG | 63 | 67 | 0.54 | 0.32-0.92 | | **0.024** | **0.034** | **0.031** |
|  | ***MTHFR*** | rs1801133 | GG | 31 | 54 | 1.00 |  | |  |  |  |
|  |  |  | GA | 43 | 63 | 0.83 | 0.46-1.51 | | 0.548 | **0.041** | 0.11 |
|  |  |  | AA | 21 | 15 | 0.40 | 0.18-0.90 | | **0.028** | 0.135 | 0.27 |
|  |  |  | A |  |  | 0.67 | 0.45-0.99 | | **0.043** | 0.061 | 0.16 |
|  |  |  | GA+AA | 64 | 78 | 0.70 | 0.40-1.22 | | 0.205 | **0.030** | 0.10 |
|  | ***KL*** | rs1207568 | GG | 68 | 97 | 1.00 |  | |  |  |  |
|  |  |  | GA | 22 | 33 | 1.04 | 0.55-1.96 | | 0.899 | 0.233 | 0.20 |
|  |  |  | AA | 2 | 3 | 1.09 | 0.18-6.78 | | 0.924 | **0.008** | **0.021** |
|  |  |  | A |  |  | 1.04 | 0.61-1.79 | | 0.879 | **0.021** | **0.023** |
|  |  |  | GA+AA | 24 | 36 | 1.05 | 0.57-1.93 | | 0.886 | 0.078 | 0.07 |
|  |  |  |  |  |  |  |  | |  |  |  |
| **Albumin**  **(37-47 g/L)** (41.70 g/L)* | ***SOD1*** | rs17880135 | TT | 196 | 210 | 1.00 |  | |  |  |  |
|  |  |  | TG | 28 | 13 | ND | ND | | ND | **0.051** | **0.013** |
|  |  |  | GG | 2 | 0 | ND | ND | | ND | 0.347 | 0.31 |
|  |  |  | G |  |  | 0.40 | 0.21-0.78 | | **0.006** | **0.032** | **0.008** |
|  |  |  | TG+GG | 30 | 13 | 0.40 | 0.20-0.80 | | **0.010** | **0.036** | **0.009** |
|  | ***SOD1*** | rs202446 | GG | 138 | 164 | 1.00 |  | |  |  |  |
|  |  |  | GT | 77 | 47 | 0.51 | 0.33-0.79 | | **0.003** | **0.003** | **0.003** |
|  |  |  | TT | 8 | 6 | 0.62 | 0.21-1.85 | | 0.390 | 0.948 | 0.67 |
|  |  |  | T |  |  | 0.60 | 0.41-0.86 | | **0.006** | **0.020** | **0.014** |
|  |  |  | GT+TT | 85 | 53 | 0.52 | 0.34-0.79 | | **0.002** | **0.005** | **0.004** |
|  | ***SOD1*** | rs1041740 | CC | 109 | 81 | 1.00 |  | |  |  |  |
|  |  |  | CT | 95 | 107 | 1.51 | 1.01-2.26 | | **0.046** | 0.199 | 0.21 |
|  |  |  | TT | 19 | 32 | 2.22 | 1.16-4.25 | | **0.015** | 0.856 | 0.62 |
|  |  |  | T |  |  | 1.50 | 1.12-2.00 | | **0.006** | 0.470 | 0.86 |
|  |  |  | CT+TT | 114 | 139 | 1.63 | 1.11-2.39 | | **0.013** | 0.253 | 0.37 |
|  | ***SOD2*** | rs4880 | AA | 75 | 90 | 1.00 |  | |  |  |  |
|  |  |  | AG | 94 | 96 | 0.78 | 0.51-1.20 | | 0.253 | 0.715 | 0.99 |
|  |  |  | GG | 59 | 43 | 0.58 | 0.35-0.96 | | **0.033** | 0.176 | 0.13 |
|  |  |  | G |  |  | 0.76 | 0.59-0.98 | | **0.032** | 0.242 | 0.17 |
|  |  |  | AG+GG | 152 | 139 | 0.70 | 0.47-1.04 | | 0.075 | 0.721 | 0.51 |
|  | ***GSTO2*** | rs156697 | AA | 67 | 80 | 1.00 |  | |  |  |  |
|  |  |  | AG | 113 | 113 | 0.86 | 0.56-1.31 | | 0.476 | 0.195 | 0.24 |
|  |  |  | GG | 48 | 31 | 0.53 | 0.30-0.93 | | **0.027** | **0.006** | **0.017** |
|  |  |  | G |  |  | 0.74 | 0.57-0.98 | | **0.037** | **0.007** | **0.019** |
|  |  |  | AG+GG | 161 | 144 | 0.76 | 0.51-1.13 | | 0.175 | **0.045** | 0.07 |
|  | ***ERCC2*** | rs171140 | CC | 67 | 44 | 1.00 |  | |  |  |  |
|  |  |  | CA | 105 | 125 | 1.94 | 1.21-3.10 | | **0.006** | **0.002** | **0.001** |
|  |  |  | AA | 53 | 56 | 1.67 | 0.97-2.88 | | 0.063 | **0.009** | **0.0036** |
|  |  |  | A |  |  | 1.29 | 0.99-1.70 | | 0.062 | **0.009** | **0.003** |
|  |  |  | CA+AA | 158 | 181 | 1.85 | 1.19-2.88 | | **0.007** | **0.001** | **0.0005** |
|  |  |  |  |  |  |  |  | |  |  |  |
| **Phosphorus**  **(0.8-1.3 mol/L)** (1.19 mil/L)* | ***IL-4*** | rs2070874 | AA | 219 | 197 | 1.00 |  | |  |  |  |
|  |  |  | AC | 63 | 86 | 1.48 | 1.01-2.16 | | **0.043** | 0.363 | 0.39 |
|  |  |  | CC | 3 | 5 | 1.94 | 0.46-8.27 | | 0.369 | 0.982 | 0.84 |
|  |  |  | C |  |  | 1.46 | 1.04-2.07 | | **0.031** | 0.419 | 0.52 |
|  |  |  | AC+CC | 66 | 91 | 1.50 | 1.03-2.18 | | **0.033** | 0.376 | 0.43 |
|  | ***ERCC4*** | rs3136166 | TT | 107 | 126 | 1.00 |  | |  |  |  |
|  |  |  | TG | 139 | 135 | 0.81 | 0.57-1.15 | | 0.241 | **0.036** | 0.10 |
|  |  |  | GG | 37 | 30 | 0.68 | 0.39-1.17 | | 0.161 | 0.198 | 0.45 |
|  |  |  | G |  |  | 0.82 | 0.64-1.05 | | 0.115 | 0.057 | 0.18 |
|  |  |  | TG+GG | 176 | 165 | 0.78 | 0.56-1.09 | | 0.151 | **0.029** | 0.10 |
|  | ***SOD1*** | rs202446 | GG | 203 | 197 | 1.00 |  | |  |  |  |
|  |  |  | GT | 74 | 72 | 0.98 | 0.67-1.43 | | 0.916 | 0.331 | 0.36 |
|  |  |  | GG | 2 | 14 | 7.09 | 1.59-31.66 | | **0.010** | **0.001** | **0.006** |
|  |  |  | G |  |  | 1.29 | 0.94-1.78 | | 0.121 | **0.011** | **0.024** |
|  |  |  | GT+GG | 76 | 86 | 1.14 | 0.79-1.65 | | 0.485 | 0.078 | 0.11 |
|  | ***GPX1*** | rs17080528 | CC | 130 | 114 | 1.00 |  | |  |  |  |
|  |  |  | CT | 133 | 138 | 1.21 | 0.85-1.72 | | 0.282 | 0.561 | 0.90 |
|  |  |  | TT | 23 | 39 | 2.01 | 1.13-3.58 | | **0.018** | 0.076 | 0.20 |
|  |  |  | T |  |  | 1.34 | 1.04-1.73 | | **0.024** | 0.113 | 0.31 |
|  |  |  | CT+TT | 156 | 177 | 1.33 | 0.95-1.86 | | 0.097 | 0.294 | 0.61 |
|  | ***GPX4*** | rs713041 | TT | 112 | 109 | 1.00 |  | |  |  |  |
|  |  |  | TC | 56 | 100 | 1.79 | 1.17-2.73 | | **0.007** | 0.070 | 0.13 |
|  |  |  | CC | 68 | 49 | 0.72 | 0.45-1.13 | | 0.152 | **0.042** | **0.04** |
|  |  |  | C |  |  | 0.91 | 0.73-1.14 | | 0.427 | 0.135 | 0.13 |
|  |  |  | TC+CC | 124 | 149 | 1.20 | 0.84-1.72 | | 0.317 | 0.925 | 0.96 |
|  | *TGFB1* | rs1800469 | GG | 110 | 122 | 1.00 |  | |  |  |  |
|  |  |  | GA | 137 | 126 | 0.83 | 0.58-1.19 | | 0.31 | 0.16 | 0.10 |
|  |  |  | AA | 33 | 39 | 1.07 | 0.63-1.83 | | 0.79 | 0.11 | **0.039** |
|  |  |  | A |  |  | 0.97 | 0.76-1.24 | | 0.83 | 0.07 | **0.023** |
|  |  |  | GA+GG | 170 | 165 | 0.88 | 0.63-1.23 | | 0.45 | 0.09 | **0.039** |
|  |  |  |  |  |  |  |  | |  |  |  |
| **Parathyroid hormone**  **(7-53 ng/L)** (123.5 ng/L)* | ***IL1A*** | rs17561 | CC | 109 | 102 | 1.00 |  | |  |  |  |
|  |  |  | CA | 85 | 77 | 0.96 | 0.64-1.46 | | 0.864 | 0.457 | 0.49 |
|  |  |  | AA | 10 | 22 | 2.32 | 1.04-5.13 | | **0.039** | **0.020** | **0.010** |
|  |  |  | A |  |  | 1.24 | 0.91-1.68 | | 0.180 | **0.043** | **0.032** |
|  |  |  | CA+AA | 95 | 99 | 1.11 | 0.75-1.64 | | 0.608 | 0.168 | 0.16 |
|  | ***IL6*** | rs1800797 | GG | 92 | 104 | 1.00 |  | |  |  |  |
|  |  |  | GA | 78 | 71 | 0.80 | 0.52-1.23 | | 0.317 | 0.259 | 0.30 |
|  |  |  | AA | 26 | 24 | 0.81 | 0.43-1.52 | | 0.511 | 0.057 | 0.058 |
|  |  |  | A |  |  | 0.87 | 0.66-1.16 | | 0.343 | **0.048** | **0.053** |
|  |  |  | GA+GG | 104 | 95 | 0.81 | 0.54-1.20 | | 0.285 | 0.097 | 0.11 |
|  | ***SHROOM3*** | rs17319721 | GG | 76 | 79 | 1.00 |  | |  |  |  |
|  |  |  | GA | 93 | 98 | 1.01 | 0.66-1.54 | | 0.980 | 0.096 | 0.11 |
|  |  |  | AA | 37 | 27 | 0.71 | 0.39-1.28 | | 0.251 | **0.023** | **0.041** |
|  |  |  | A |  |  | 0.88 | 0.66-1.16 | | 0.348 | **0.016** | **0.027** |
|  |  |  | GA+AA | 130 | 125 | 0.92 | 0.62-1.37 | | 0.687 | **0.031** | **0.044** |
|  | ***UMOD*** | rs12917707 | GG | 133 | 143 | 1.00 |  | |  |  |  |
|  |  |  | GT | 67 | 49 | 0.69 | 0.44-1.06 | | 0.091 | **0.015** | **0.015** |
|  |  |  | TT | 3 | 6 | 1.87 | 0.46-7.66 | | 0.382 | 0.403 | 0.32 |
|  |  |  | T |  |  | 0.83 | 0.57-1.21 | | 0.336 | **0.016** | **0.013** |
|  |  |  | GT+TT | 70 | 55 | 0.74 | 0.48-1.13 | | 0.158 | **0.012** | **0.011** |
|  | ***ICAM-1*** | rs5498 | AA | 41 | 63 | 1.00 |  | |  |  |  |
|  |  |  | AG | 100 | 97 | 0.64 | 0.39-1.04 | | 0.069 | 0.436 | 0.42 |
|  |  |  | GG | 66 | 47 | 0.45 | 0.26-0.78 | | **0.005** | 0.138 | 0.16 |
|  |  |  | T |  |  | 0.67 | 0.51-0.89 | | **0.005** | 0.137 | 0.16 |
|  |  |  | AG+GG | 166 | 144 | 0.56 | 0.36-0.89 | | **0.014** | 0.239 | 0.24 |
|  |  |  |  |  |  |  |  | |  |  |  |
| **C-reactive protein**  **(<10mg/L)** (10 mg/L)** | ***SOD2*** | rs4880 | AA | 91 | 40 | 1.00 |  | |  |  |  |
|  |  |  | AG | 122 | 30 | 0.52 | 0.30-0.91 | | **0.022** | 0.291 | 0.27 |
|  |  |  | GG | 64 | 16 | 0.54 | 0.28-1.06 | | 0.071 | 0.331 | 0.32 |
|  |  |  | G |  |  | 0.70 | 0.50-0.98 | | **0.035** | 0.285 | 0.27 |
|  |  |  | AG+GG | 186 | 46 | 0.53 | 0.32-0.87 | | **0.013** | 0.233 | 0.22 |
|  | ***GSTP1*** | rs749174 | GG | 117 | 44 | 1.00 |  | |  |  |  |
|  |  |  | GA | 118 | 35 | 0.78 | 0.47-1.31 | | 0.352 | 0.706 | 0.78 |
|  |  |  | AA | 43 | 6 | 0.36 | 0.14-0.91 | | **0.031** | 0.883 | 0.91 |
|  |  |  | A |  |  | 0.67 | 0.46-0.97 | | **0.033** | 0.972 | 0.98 |
|  |  |  | GA+AA | 161 | 41 | 0.67 | 0.41-1.09 | | 0.108 | 0.803 | 0.86 |
|  | ***XRCC1*** | rs25487 | CC | 116 | 29 | 1.00 |  | |  |  |  |
|  |  |  | CT | 120 | 36 | 1.21 | 0.70-2.12 | | 0.495 | 0.163 | 0.15 |
|  |  |  | TT | 38 | 19 | 2.03 | 1.02-4.04 | | **0.045** | 0.480 | 0.50 |
|  |  |  | T |  |  | 1.39 | 0.99-1.96 | | 0.058 | 0.297 | 0.31 |
|  |  |  | CT+TT | 158 | 55 | 1.41 | 0.84-2.36 | | 0.189 | 0.171 | 0.16 |
| **Ferritin**  **(25-250 (ug/L)** (154.5 ug/L)* | ***SOD2*** | rs4880 | AA | 50 | 59 | 1.00 |  | |  |  |  |
|  |  |  | AG | 72 | 65 | 0.73 | 0.44-1.22 | | 0.235 | **0.020** | **0.023** |
|  |  |  | GG | 35 | 35 | 0.82 | 0.45-1.51 | | 0.527 | 0.058 | 0.056 |
|  |  |  | G |  |  | 0.88 | 0.66-1.20 | | 0.441 | **0.035** | **0.035** |
|  |  |  | AG+GG | 107 | 100 | 0.76 | 0.48-1.22 | | 0.259 | **0.012** | **0.013** |
|  | ***GSTP1*** | rs749174 | GG | 65 | 78 | 1.00 |  | |  |  |  |
|  |  |  | GA | 70 | 64 | 0.76 | 0.47-1.23 | | 0.261 | **0.027** | **0.023** |
|  |  |  | AA | 22 | 20 | 0.76 | 0.38-1.52 | | 0.429 | 0.103 | 0.09 |
|  |  |  | A |  |  | 0.83 | 0.61-1.16 | | 0.280 | **0.029** | **0.024** |
|  |  |  | GA+AA | 92 | 84 | 0.76 | 0.49-1.19 | | 0.228 | **0.016** | **0.013** |
|  | ***SLC7A9*** | rs12460876 | TT | 47 | 64 | 1.00 |  | |  |  |  |
|  |  |  | TC | 89 | 71 | 0.54 | 0.33-0.89 | | **0.016** | **0.005** | **0.006** |
|  |  |  | CC | 21 | 26 | 0.82 | 0.41-1.66 | | 0.584 | 0.756 | 0.93 |
|  |  |  | C |  |  | 0.81 | 0.58-1.13 | | 0.216 | 0.242 | 0.30 |
|  |  |  | TC+CC | 110 | 97 | 0.59 | 0.37-0.96 | | 0.032 | **0.017** | **0.022** |
|  | ***GPX4*** | rs713041 | TT | 65 | 49 | 1.00 |  | |  |  |  |
|  |  |  | TC | 45 | 60 | 1.87 | 1.09-3.22 | | **0.024** | 0.084 | 0.08 |
|  |  |  | CC | 28 | 32 | 1.58 | 0.84-2.99 | | 0.157 | 0.632 | 0.60 |
|  |  |  | C |  |  | 1.32 | 0.97-1.81 | | 0.082 | 0.428 | 0.40 |
|  |  |  | TC+CC | 73 | 92 | 1.76 | 1.08-2.87 | | **0.023** | 0.147 | 0.14 |

*Median as a cut-off, **normal value as a cut-off in the logistic regression analysis; Group 1: individuals with low-risk biochemical parameter; Group 2: individuals with high-risk biochemical parameter indicative of poor prognosis. ND, No data. **^#^**ORs and the corresponding 95% CIs were adjusted for age and gender.
